# Food justice: processes, practices and perspectives

**DOI:** 10.1007/s41130-023-00188-4

**Published:** 2023-02-01

**Authors:** Camille Hochedez

**Affiliations:** Université de Poitiers – UMR Migrinter, Poitiers, France

In 2016, we published a special issue of the online journal *Justice spatiale* | *spatial justice* entitled ‘Food Justice and Agriculture’ (Hochedez & Le Gall, 2016). One of the issue’s major themes was to examine in greater depth the notion of food justice and to set it in the field of Francophone geography by placing agriculture at the heart of its definition. We called for a more *agri*food-based and spatially based justice. The other theme was to reflect on the processes, in particularly related to agriculture, that lead to food justice. Six years later, with this special issue, we want to build on the achievements of the JSSJ volume to introduce complexity into the analysis of food systems through the lens of agrifood justice.

This themed issue can be seen in a dual context, which has changed since 2016. First and foremost, on a global scale, discussing food and farming is even more meaningful today. Food insecurity continues to grow, and has become even more visible with the geopolitical context (war in Ukraine) and the health context (COVID-19 pandemic since 2020). Rapidly rising food prices reflect the interdependencies between stakeholders and spaces in food systems, their globalised structure, relationships of domination and the power of middlemen (large-scale distribution, stock exchanges, etc.). These trends have repercussions on every level, especially for the most precarious populations for whom it is even more difficult to feed themselves properly. One example is the food insecurity of French students during the COVID-19 crisis, who had no access to the subsidised collective restaurants they used in normal times. Food injustices were, then, accentuated in light of this global context. And then on a local level in several western countries, the institutionalisation of food as a tool of governance of transitions in areas became the stage to spread the ‘sister’ notions of food justice in food policies, along with ‘food democracy’ and ‘food solidarity’. These notions often constitute the social axis of food policies such as the PAT (Projets Alimentaires Territoriaux) in France (Pahun & Fouilleux, 2022). They represent one way of taking food inequalities into account, even if PATs often reduce the issue to a matter of food accessibility. It is worth looking at the extent to which these policy tools include a systemic approach to food justice when designed.

These changes validate our aim to take a further look at the conceptualisation of food justice, which, inevitably, we still consider as *agri*food justice. The articles in this special issue contribute to this aim, and highlight the complexification of relations between food systems’ various elements as well as in the way in which they are considered (Clouette, 2022; Darrot and Gallardo, 2022; Guillemin, 2022; Proust, 2022; Sosa Varrotti et al., 2022). The approach through complexity, and the introduction of nuances in agrifood justice thinking, was a wish expressed in the special issue’s call for papers with its three ambitions. First, this involved establishing the procedural and practical approach of the notion of food justice in reaction to the classic approach based only on food accessibility. The aim was then to put food systems back into social systems, in interaction with a planetary ecosystem subject to global changes. Lastly, we wished to broaden the meaning of agrifood justice to include emerging but as yet little-studied questions, especially by exploring the intermediary spaces of processing, transport, logistics and distribution, or by examining the role of stakeholders in agricultural and commercial entrepreneurship, education or the arts.

If viewed in a cross-sectional way, the issue’s five contributions do not fully meet these objectives. The fact that the articles are rooted in agricultural spaces is an achievement compared with the 2016 journal issue. It shows the need to put agricultural and production resources back at the centre of the discussion on food injustices. However, the authors have appropriated the reference framework of agrifood justice to varying degrees. It is sometimes added almost as an afterthought because it was not the theoretical basis for their study. Furthermore, most of the articles are written by French scientists who, even if working in Southern countries, reason according to the frame of reference of inequalities, a legacy of the French social geography tradition, or use a Bourdieusian approach to the social space. We should point out the almost total lack of articles from foreign researchers (with the exception of a single proposal from Argentinean colleagues): although the journal is published in English, above all, we have the point of view of French-speaking scientists, which could, moreover, explain the difficulties of using the agrifood justice frame of reference to think about food systems. What’s more, although the call for articles suggested exploring 4 themes (‘mechanisms of injustice and social vulnerabilities at all levels of agrifood systems’, ‘justices and injustices in the intermediate spaces of food systems’, ‘processes rooted in an ecosystem of the Earth subject to global changes’ and ‘How to achieve a just food transition?’), the articles make contributions on some themes (social vulnerability, intermediary spaces) while others are little- or un-explored, or even overlooked (empowerment, the role of art and education, the links between food justice and environmental justice through exploring the exploitation of natural resources by food systems).

Nevertheless, even though some themes are not addressed head-on, these elements of analysis provide a more detailed and nuanced reading of situations. For example, although the issue of violence in food systems is not dealt with directly, the analysis in terms of relations of domination or power by certain authors (Guillemin, Proust, Clouette) offers food for thought on the matter by introducing more detailed elements than the now well-documented problems of suicides in the agricultural world (Deffontaines, 2020; Jacques-Jouvenot, 2014) or the expulsion of indigenous populations from the land they farm (Grajales, 2021; Khairina & Lund, 2020; see reviews of these two studies following the special issue articles). The symbolic, sometimes hidden ‘small acts of violence’, which can take the form of material violence (economic, social, etc.—see the question of cooperatives fixing the price of market garden produce in Guillemin’s article), are, in reality, major acts of violence and give tangible form to ‘peasant suffering’ (Deffontaines, 2014). In line with these ‘small acts of violence’, some authors examine the perception of injustice through the issue of representations: for example, socially constructed representations of ‘good’ and ‘bad’ fish (Clouette), the perception of injustice linked to social trajectories (Guillemin), or the downgrading and reclassification of farming families (Sosa et al*.*). In passing, these three texts highlight the fact that power relations are not only structural, and this calls for a procedural approach to agrifood justice.

Although this issue cannot answer all the questions it raised in its call for articles, it brings together papers that make notable contributions to understanding agrifood justice, which we present in two stages. First, we present the way in which the articles appropriate the question of social injustices inherent in food systems and reposition them in the context of a changing global ecosystem. The other contribution that we develop is the procedural approach of agrifood justice both in the way that injustices are seen as a process of disconnection between different dimensions of agrifood systems and how justice actions are conceived of as a process of inclusion.

## Food injustices embedded in social spaces that are themselves rooted in a changing global ecosystem

By focusing on food systems’ stakeholders, first and foremost the farmers and production and processing middlemen, the articles shed light on the social injustices at the root of food injustices from the perspective of more or less symbolic violence that reflects asymmetrical relationships between actors. These relationships are a source of vulnerability for some and of power for the others. Rather than a reading in terms of dominant and dominated, these articles offer a nuanced interpretation of the power relations at the heart of injustices, in particular by illuminating them with a dynamic reading that takes into account the trajectories of individuals as well as by anchoring them in changing ecosystems.

### Power relationships—the very heart of agrifood injustices

All the articles offer an implicit reflection on the power relationships and mechanisms of domination underlying food injustices, thus shedding light on the mechanisms that produce social vulnerabilities, as well as on more invisible but no less violent forms of domination. The texts also provide nuance in their analysis of the social worlds of food, being situated beyond a classist interpretation. They scrutinise the ‘grey areas’ of how food systems operate, which cannot be reduced to an opposition between ‘good’ and ‘bad’ (Clouette), or between ‘just’ and ‘unjust’.

It is striking to note that many papers document the power relationships inherent in agricultural production according to a Bourdieusian reading of the social spaces of food. They are considered sometimes only from the consumers’ point of view (Gallardo Gomez & Darrot), sometimes in the links between producers and consumers, and sometimes only from the producers’ point of view (Sosa et al.). Whether focused on ‘social worlds’ and ‘social fractions’ (Guillemin), or on the ‘social profiles’ of producers (Proust), these approaches are a way of examining the social inequalities that underpin food production. The expression ‘social worlds’ (Guillemin) is a way of not reducing relationships to merely the dominant/dominated opposition, but also revealing their complexity. In this respect, the authors use different terms to describe the social fractures that pervade food systems, reflecting different ways of analysing these social worlds. The opposition is relatively binary for some of them: rich versus poor, and industrial fisher versus small-scale artisanal fishers in Clouette’s article, and focusing on ‘low-income populations’ for Gallardo Gomez and Darrot. The complexity of agricultural social worlds is analysed by Proust in São Paulo: she points out the different social profiles of gardeners and farmers. It is expressed by Guillemin in terms of ‘social class fractions’: the author identifies five ‘fractions’ among the group of market gardeners in Normandy. This is a way of breaking away from the uniform vision of the ‘little market gardener’ and, on the contrary, underlining the social heterogeneity of the sector. The complexity is also present in Sosa et al.’s study of land grabbing in Argentina, which shows the diversity of the profiles of farmers involved in the process: family farmers, agribusiness enterprises and multinational companies.

Several articles, therefore, introduce nuance into the reading of vulnerable populations involved in the process of agricultural production so that the reading of the social worlds of food systems does not merely focus on poor or racialised populations but encompasses intermediate situations of insecurity (e.g. the ‘impoverished agricultural fractions’ analysed by Guillemin). This is a way of responding to the conclusive injunction formulated in the introduction to the 2016 issue of JSSJ, in particular ‘the urgency of deciphering the power relations taking place via agricultural and food resource’ (Hochedez & Le Gall, 2016).

Ultimately, in this issue’s articles, the place taken by the analysis of power relations at work in the social worlds of food is another way of saying that social injustices represent the matrix of food injustices, echoing the still relevant analysis by Slocum and Cadieux according to which ‘true food security is impossible without social justice being understood as one of the necessary starting points for analyses of, and solutions to, food insecurity’ (Slocum et al., 2016, p. 3). In this sense, Proust’s reading of the socio-spatial dynamics of urban agriculture in São Paulo is especially interesting. She interprets the distribution of different forms of urban agriculture according to the social classes that practise it, showing that the duality of the forms of agriculture (amateur gardening and professional subsistence agriculture) follows the socio-spatial divisions of the city, i.e. the patterns of urban segregation: gardens are located in the centre, while subsistence farming is pushed out to the margins. In the Brazilian context, she also shows that this matrix of urban socio-spatial injustices is superimposed by racial inequalities, which play a role in the way people eat, but also in unequal access to farmland. This is a way of taking a fresh look at the concept of food apartheid (Brones, 2018; Washington, 2021), describing a differentiated access to food depending on one’s ethnic background. Proust broadens the conception of food apartheid, traditionally thought of as a racialised food desert, showing that it is not only a matter of access to quality food, but also of access to land. According to Proust, the urban agricultural dynamics at work in São Paulo do not allow for a ‘deracialisation of access to land and food’.

### Four productive system ‘nodes’ at the heart of power relations

The articles do not merely observe the existence of asymmetric power relations in food systems. Their interest lies in examining the basis of these relationships. In the issue of JSSJ that we coordinated, Slocum et al. (2016) identify four ‘nodes’ (trauma/equity, exchange, land and labour) on which to concentrate changes towards greater food justice (these are ‘areas, finally, around which to build solidarity’ into the food system). Each in their own way, the articles shed light on these four nodes at the heart of power relations, at least three of which overlap with those identified by Slocum et al.

First, land seems to be the cornerstone of food injustice because it is a materiality at the root of power relations, corresponding to the ‘land’ node identified by Slocum et al. (2016). In this, the articles respond to the research proposal we formulated in 2016 to examine ‘injustice linked to land-access, which is urgently needed to preserve equitable access to resources’. Implicitly, they shed light on the land dimension of agrifood justice, which some (Baysse-Lainé & Perrin, 2021) isolate through the notion of land justice. This represents an ‘evaluation grid allowing specific attention to be paid to power relations and social, gender or ethnic/religious inequalities’ (Baysse-Lainé & Perrin, 2021). This affirms that land is indeed at the heart of power relations and that relationships of domination are established there.

The texts shed new light on land justice in comparison with French-language studies, which tend to focus on public regulation mechanisms and planning issues (Baysse-Lainé, 2018; Perrin & Nougarèdes, 2020), because as well as examining public regulation mechanisms (Proust), they also look at private regulations (Sosa et al.). Proust’s article examines the mechanisms of domination through land in several ways. First of all, it shows that the location and quality of cultivated land corresponds to a social hierarchy and relationships of domination that overlap with racial and gender inequalities. The subsistence farming practised by impoverished populations in the interstices corresponds to land that is poor and less suitable for agriculture. This converges with one of the conclusions reached by Baysse-Lainé and Perrin about the qualitative dimension of unequal access to land between farmers: some marginal or marginalised groups of farmers are relegated to the less productive margins of the agricultural space (Baysse-Lainé & Perrin, 2021). Next, Proust shows that relationships of domination through land are also exercised through the control of access to land by certain actors against a background of violence and crime: drug cartels and the police, but also public policies from the State that encourage a ‘laissez-faire’ attitude in the town-planning process for the outskirts. The mechanisms of land allocation are, then, linked to urban speculation. Ultimately, land is a tool of the social reproduction of urban inequalities. In their paper, Sosa et al. consider the private stakeholder in the form of foreign companies that invest by purchasing agricultural land in Argentina (land grabbing): the participation of capital or foreign stakeholders in agricultural property is a symbol of the relationships of domination in productive systems, and represents a form of economic and symbolic violence. However, this article also sets land-related injustices in the long history of land ownership in the context of a ‘new country’, illuminating the traumatic dynamics of land justice (Baysse-Lainé & Perrin, 2021). The process of land grabbing takes place to a backdrop of already unevenly distributed land structures. Land inheritance establishes leasing and purchasing land in situations of injustice built on the very long term. Companies lease and then return land at the discretion of very unstable granted concessions and lease contracts. The articles therefore examine food justice via the land node without, however, making land justice as such essential. It is a question of considering land as one of the dimensions of agrifood injustices, as a tool of the mechanisms of domination (Horst et al., 2021), beyond a strict understanding of land justice as ‘equitable and inclusive access to land, to the resources it contains and to the building rights connected to it’ (Baysse-Lainé & Perrin, 2021). The texts suggest moving beyond an approach in terms of accessibility by taking into consideration other injustices linked to farmland (material and symbolic violence, farmers’ perceptions of injustice, the traumatic dimension linked to the racial context, etc.).

Second, the articles offer more or less directly a discussion about labour, which corresponds to the fourth node of food justice identified by Slocum et al. (2016). The attention paid to working conditions in agricultural productive systems comes in response to the research avenue we formulated in 2016, namely ‘conceiving agricultural resources also from the point of view of minorities and marginalised persons’. The texts shed light on forms of creating hierarchies in agricultural labour, notably by analysing the presence of marginalised groups in productive activities. Guillemin’s article highlights the various forms of working relationships depending on the social position of the producers, which constitute different economic models. The ‘agricultural bourgeoisie’ in Normandy is thus part of a logic of agrarian capitalism when hiring seasonal workers, sometimes of foreign origin, in a logic of seeking labour flexibility. The presence of different farmworker statuses produces a social hierarchy on farms. Some texts also show how agricultural labour can be an economic resource for precarious groups (unemployed people for Guillemin, women and ethnic minorities for Proust) so that market gardening represents both an economic opportunity and a tool of social reclassification. The interest of these analyses is to show how, while still part of hierarchical relationships, agriculture does not help reduce situations of injustice, but moves the lines of the agricultural social worlds by participating in trajectories of social ascension or “reclassification” (Guillemin). For example, agricultural alternatives (short food supply chains, organic farming) can help to provide work and income-generating opportunities for formerly unemployed people from non-agricultural professional backgrounds without, however, ‘addressing the structural inequalities across the farming community, particularly in retirement’ (Guillemin). For Clouette, they also contribute to enhancing the value of less sought-after species of fish (the Poiscaille system along the lines of vegetable boxes, or ‘*godaille*’) and at the same time providing additional income for sailors. This echoes other studies on farming as a resource in anchoring migrants (Darly et al., 2021; Hochedez & Lessault, 2021). Agricultural labour therefore represents a node of food justice in the sense of an entry point for solidarities from which potentially just agrifood spaces develop (Slocum et al., 2016): it has both an alienating dimension constitutive of power relations on farms, but can also provide forms of social emancipation.

A third way in which the articles shed light on the bases of power relations in agrifood social worlds lies in the analysis of the role of middlemen (farming cooperatives, marketing networks, etc.). Some authors underline that relationships of domination, and therefore the hierarchy of agricultural social worlds, are also based on the power held by certain groups in the intermediary spaces of food systems. Using the example of vegetable growers’ cooperatives, Guillemin shows that the agricultural bourgeoisie occupies positions of power in cooperatives while remaining obedient to orders from above. The domination is exercised here through the position of power they hold in agrifood management. This is especially visible in price-setting mechanisms—the cooperatives’ managers still set prices, which leads to forms of standardising practices (for example in the use of phytosanitary products) in order to get a better price for vegetables. Both national and foreign agrifood businesses are another major intermediary playing a role in the mechanisms of domination. Sosa et al. analyse key land-grabbing players in Argentina (in the provinces of Chaco and Misiones) by raising the question of foreign investments in farmland. In 2015, 6.09% of Argentina’s national territory was in foreign hands, and the rate is over 50% in some provinces such as Neuquén. The authors show that land grabbing is carried out by both national agrifood companies described as ‘mega companies’ that also have the capital to invest in agricultural property in neighbouring Mercosur countries (they control over 200,000 ha in these countries, either by leasing or purchasing land), and also foreign companies (Louis Dreyfus, etc.) that invest using various financial arrangements (hedge funds, pension funds, private equity funds). In fact, middlemen shape the spaces and moments when the power relations of food systems are forged. This is how Clouette analyses the ‘*débarque*’ or ‘landing’ seen as a food space–time including both the killing and marketing of fish. This space–time is intermediary in two respects. On the one hand, it crystallises the construction of the symbolic quality of the fish, especially by establishing the difference between ‘high’ and ‘low’ quality fish. On the other hand, the ‘landing’ is a space of asymmetric exchange between fishers and middlemen, where a price is set that is not always ‘just’. In these power relations, it is above all industrial fishing companies that benefit from gains, at the expense of small-scale enterprises.

The fourth node of relationships of domination highlighted in these articles involves the economic dimension, whether this is through the question of price setting, costs or more generally economic mechanisms on a large or small scale. This echoes the third node of exchange, identified by Slocum et al. (2016). This goes far beyond the question of price setting and ‘just price’. In some articles, financial capital thus appears as a negotiating tool that contributes to relationships of domination in the same way as land or labour. Financial capital is an instrument that makes it possible to negotiate the other nodes, to force the issue, since some stakeholders have the capacity to negotiate and others do not. This is what Sosa et al. describe when they analyse the outflow or inflow of investors in the agricultural land market, which may be supported by price policies on certain commodities or by fiscal policies.

The articles raise a final interesting point. They analyse the consequences of relationships of domination in each of food justice’s nodes. For example, they show how these nodes influence the perception of the quality of food products, which leads to repercussions for purchases and marketing methods. Clouette argues that in the fishing industry, what makes a fish ‘beautiful’ or ‘ugly’ depends more on the way the fish was caught, i.e. the quality of the fisher’s work, than on organoleptic properties. The quality of the fish is the result of a process: what makes it high quality is also the fishing technique used and the marketing network. For Guillemin, the quality of a vegetable depends on the line of phytosanitary treatments: the example of the differentiated flow of treated and untreated leeks introduces health and environmental considerations into the perception of food quality. This is connected to relations of domination in the productive sphere. Clouette shows that the binary division between ‘beautiful’ and ‘ugly’ fish intersects the fault line between small-scale fishing that produces a ‘well caught’ fish and industrial fishing, as well as a social fault line among consumers, between urban Parisian consumers who can afford to buy ‘high quality’ fish and the working classes living on the outskirts of cities or near landing zones who are more likely to eat ‘ugly’ fish, with the exception of ‘*godaille*’.

### A fledgling reflection on productive social worlds as part of the global ecosystem

The third aspect of this issue’s call for papers lies in the environmental dimension of food justice. We wanted to analyse ‘processes of agrifood injustices rooted in an ecosystem of the Earth subject to global changes’. Several avenues were suggested such as human/non-human relations, means to adapt to climate change and access to resources in the reduction or exacerbation of food injustice. It has to be said that from this point of view, this special issue does not fulfil its objective: it does not address head-on the links between agrifood justice and the environment. However, several articles point out some environmental dimension of agrifood justice in a more indirect way, through three aspects.

The first involves agricultural or fishing production modes as more or less respectful of the global ecosystem, and more or less connected to it. One obviously thinks of organic methods and the use of phytosanitary products (Guillemin) or the consumption of organically certified produce (Gallardo Gomez & Darrot). In a more original way, Clouette sees human/non-human relations as essential to defining food ethics and setting standards, via the techniques of killing and carving fish, which are more or less respectful of the fish. The author discusses *ikejime*, a technique believed to guarantee as little suffering as possible for the animal so therefore more socially acceptable. Techniques such as these participate in the definition of ‘good food’ (both good quality and good on an ethical level, setting standards for what is ‘good’ and ‘bad’). ‘Care’, in the sense of the care taken of the animals, is, therefore, part of the definition of ‘food morals’.

The second deals with the use of natural resources, an issue that Clouette examines in depth. He shows that the relationship with the world ocean and the use of this resource are a criterion for differentiating the quality of fish. The fishing method (fishing techniques, overfishing vs. artisanal fishing) is a criterion for distinguishing between decried industrial fishing, responsible for the depletion of certain species, and artisanal fishing, which is more sustainable. Artisanal, smaller-scale fishers seem, then, to guarantee respect for nature and the seasonality of species. The text thus makes it possible to put forward the idea that a more sustainable use of resources is a criterion for defining fairer agrifood practices.

Although the environmental aspects are not truly conceptualised as elements feeding into the theory of agrifood justice, this special issue nevertheless shows how the environment is a component and even a major stake in the power relations at work in food systems, in other words, it is the medium for asymmetric power relations. Some of the articles highlight the practice of greenwashing, which, in these cases, consists in those who dominate food systems manipulating environmental arguments despite their activities being mostly prejudicial to the global ecosystem. Guillemin shows that vegetable-growers’ cooperatives use ‘residue-free’ practices to devalue certain producers, which actually generates new agrifood injustices among vegetable growers. He develops the case of a large-scale vegetable producer who feels he has been ‘screwed’ by wanting to reduce pesticide use on his leeks affected by thrips (insects that ravage the green part of the vegetable) in the hope of developing more sustainable practices. However, the cooperative does not enhance their value in the price they pay for these leeks, even though leek thrips do not prevent the vegetable’s consumption. On the contrary, his produce is declassified by a lower purchase price so that this farmer’s environmental practices work against him and translate as a drop in income for his farm amounting to €3000 per hectare. Via the intermediary of lower remuneration that does not encourage environmental practices, the managers of farming cooperatives exercise a relationship of domination over vegetable growers, including those who are well established in the vegetable production landscape. The study by Sosa et al. addresses the issue of greenwashing directly, showing how the discourse of companies involved in the process of land grabbing, notable for their inclination towards large, mechanised and polluting crops (e.g. rice and soya) promotes farming practices that are presented as sustainable (introduction of rotations between rice and fish crops that kill bacteria).

Indirectly, the articles that focus on the agricultural dimension of food justice consider the links between agricultural production and the global ecosystem as a dimension of the power relations at play in food systems. This does not mean isolating the environment as a factor of injustice per se but instead incorporating it into a systemic reflection about factors of disconnection leading to situations of injustice. In doing so, these discussions are part of a procedural approach to agrifood justice. In other words, they consider situations of injustice as the result of connections or disconnections between the different elements of food systems.

## Agrifood justice as a process

This special issue aims to establish a procedural approach to agrifood justice, which leads us to define it as ‘a process that (re)connects all food systems’ activities, spaces, actors and stakeholders in order to make them more inclusive, at the level of all their components (how food is produced, transported, processed, marketed, distributed, consumed, digested, designed and considered)’ (Hochedez & Le Gall, 2018). According to our approach, agrifood justice is not so much an ideal to achieve as a process of reconnection between the different components of food systems considered on every level (from the body to the planet), in a broad way (from the soil to the stomach and the composter), in their material and immaterial dimensions (land, networks, representations, policies) from an interdisciplinary perspective (life sciences, experimental, social/arts) and a relational approach. The articles feed into this approach, presenting a certain number of contributions on various processes of (dis)connection between elements of food systems.

### Agrifood injustice as a result of disconnections between food systems’ dimensions and actors

The articles analyse the processes and mechanisms that lead to situations of disconnection and, ultimately, to the creation of situations of injustice. They propose a dynamic approach to agrifood justice.

Some of the articles (Sosa et al., Proust and Guillemin) shed light on the way in which situations of agrifood injustice arise from a process of capitalist accumulation on the one hand and the neoliberalisation of agriculture on the other, referring to the forms of development of ‘agricultural capitalism’ (Tsing, 2009). Proust’s study highlights the mechanisms of land allocation in connection with urban speculation. In doing so, she shows that urban agriculture participates in the process of financialising the city and the dynamics of building a neoliberal city. In this way, she converges with the critical analyses of urban agriculture that sometimes denounce its lack of social inclusiveness, sometimes its participation in ‘urban strategies [that] may mainly benefit the property class and newcomers rather than disadvantaged communities’ (Horst et al., 2017, p. 278), thus reinforcing the segregative urban processes (e.g. gentrification) that are already at play (Tornaghi, 2014). This is also what Paddeu underlines when she shows the extent to which urban agriculture is rooted in the logics of ‘green capitalism’, in other words that certain new forms of urban agriculture lead to the perpetuation of a model of capitalist development (Paddeu, 2021). This special issue broadens the analysis beyond the framework of urban agriculture to encompass forms of rural capitalist agriculture. Sosa et al. show that the injustices linked to the process of land grabbing are the result of a process of accumulating land, economic and financial capital. Land grabbing is thus part of a process of financialising agriculture that is both based on and results in the intensive use of capital to control agricultural resources. In this process, land becomes a financial asset, making it possible to accumulate increasing income for groups that are already formed. Farmland becomes a speculative investment via various tools such as futures markets or a range of other highly sophisticated financial instruments (Guibert et al., 2015). Ultimately, farmland is at the heart of a process of the social reproduction of injustices in cities and countryside alike.

Other studies (Gallardo Gomez & Darrot, Clouette) consider the processes of creating agrifood injustices by analysing the mechanisms that contribute to disconnections between producers and eaters. In this process, agrifood injustices are indeed constructions based on representations of eaters or producers. Clouette shows that consumption inequalities are a function of how producers (fishers) are viewed. They represent the starting point for a discussion about the effects on the productive sector, which is a way of taking into account the material and symbolic (dis)connections between fishers and consumers. In this sense, Clouette takes a fresh look at the notion of ‘food desert’, traditionally used to spatialise the inequalities of food accessibility, by applying it to the fishing industry: he suggests the notion of ‘fish desert’ or ‘seafood desert’, a space more or less superimposed on the more classic food deserts, and more likely to concern consumers on the outskirts of Paris as well as, paradoxically, residents of coastal areas. He relativises the idea that physical proximity to the sea offers better access to fish produce. Indeed, access to fish depends more on access to marketing networks and therefore relational proximity than spatial proximity. To circumvent fish deserts, Clouette shows that there exist mechanisms such as the development of informal networks by small-scale fishers that benefit the working classes who, without this, could not consume fish (unlike people from urban areas) despite living in proximity to ports. The analysis confirms that (dis)connections depend more on inter-acquaintance links between producers and eaters than on the physical proximity between them, which is not enough. In the same way, Gallardo Gomez’ and Darrot’s analysis of two food transition initiatives (access to organic produce in a social grocery and an ‘*AMAP*’ or association to support peasant farming) from the point of view of consumers in situations of financial precarity aims to include the issue of food accessibility in a systemic vision of food. Although the reference framework of sustainable development is chosen over that of agrifood justice, the study nevertheless insists on social inclusion and participation as conditions of connection between precarious consumers and organic producers. However, the authors show that both initiatives fail to improve the participation and social inclusion of these populations. One of the reasons for this lies in the fact that these actions are not systemic but instead mostly focused on consumption, hence the (dis)connections between various dimensions of agrifood systems that are not dealt with, to the detriment of a focus on the consumption aspect through the issue of accessibility, democracy or food vulnerability.

Ultimately, all the studies show that situations of injustice are not a given, nor are they set in stone or irreversible, but that they are the result of processes. They shed light on the mechanisms (land, social, economic, etc.) that lead to disconnecting consumers from producers, or disconnecting the various components of food systems from one another. The mechanisms they describe also shed light on the processes and actors that lead to more justice, in response to the question asked by Slocum and Cadieux: ‘what does it mean to do food justice?’ (2016).

### Agrifood justice as a process of inclusion

Whereas classic approaches to justice highlight the fractures and structural exclusions that pervade our societies and ecosystems via issues of networking and accessibility, this special issue favours an approach via connections, opening up the range to less ‘extreme’ situations of exclusion, which also leaves room for adaptations.

From this perspective, the articles offer a reading of justice as a process of including certain minoritised social groups in local agrifood systems. Whether this is through the prism of women and racialised minorities in Brazil (Proust), precarious populations from the ‘Gilets Jaunes’ (Yellow Vests) movement (Guillemin) or low-income populations (Gallardo Gomez & Darrot), the authors examine the way in which these minority social groups, traditionally excluded from local food systems, are gradually participating, to varying degrees, in the dynamics of relocalising production and consumption. This inclusion process is sometimes accompanied by a change of the social positions of minorities according to a trend of ascension thanks to agricultural activity (Guillemin, Proust). These analyses intersect with the hypothesis that, to be just, local food systems should depend on ‘the ability of immigrants and refugees, people of colour, low-income communities (…) to produce, access, and consume healthy and culturally appropriate foods’ (Agyeman, 2013, p. 136). The interest of the articles lies in critically deconstructing this process of minority inclusion towards greater food justice.

The medium for this process of inclusion often lies in the figure of the food initiative, defined as a ‘real or projected achievement, using different resources (human, technical, financial) and actors, to reach an objective’ (Soulard & Duvernoy, 2017). Whether this is through urban agriculture initiatives (Proust), food relocalisation for underprivileged populations (Gallardo Gomez & Darrot, Clouette) or proximity agriculture (Guillemin), these articles are worth reading because they present these initiatives not as objects in themselves but as processes. What the studies by Proust and Gallardo Gomez and Darrot have in common is examining empowerment as the cornerstone of the process to include marginalised populations. Social inclusion and empowerment are very closely linked in that they tend towards the same movement to transform the ‘geometry of power’ (Hochedez & Le Gall, 2016) within agrifood systems. Empowerment is the process of an individual’s or a collective’s autonomy by increasing their knowledge and their capacity for action (Bacqué & Biewiener, 2015). Above all, it depends on a learning process. From this point of view, Proust offers interesting thoughts on what it means to ‘do food justice’ through urban agriculture. Admittedly, urban agriculture initiatives are not food justice initiatives because they emphasise certain functions of urban agriculture at the expense of others. In this, they are more akin to food sovereignty or food security. However, where they can build spaces of agrifood justice for some marginalised groups is in their capacity to be the medium for a process of empowerment, especially for women, which Proust analyses using the frameworks of eco-feminism. Urban agriculture thus takes on a political dimension with regard to certain groups. In doing so, it is not only a productive activity but also and above all a ‘daily political mobilisation for subsistence’ (Pruvost, 2021).

Via the range of initiatives described, the main mechanism for minority inclusion is that of giving or having ‘the right to have access to’ high quality food (Gallardo Gomez & Darrot), and means of production (Guillemin, Proust), especially land (Proust, Sosa). Proust’s article focuses on access to land, considered as the first milestone in one’s right to produce one’s own food. This analysis aligns with a procedural approach to agrifood justice since it is in line with that of relationships with land, seen as ‘necessary foundations for rebuilding a new civilization and a new humanity’ proposed by Safransky when she talks about land justice (2018, in Baysse-Lainé & Perrin, 2021). This approach is a way of taking a fresh look at the notion of accessibility, which cannot be reduced to a passive process where minorities receive but rather one in which they also have to power to act to improve their own access to food and the means to produce it. In this, the ‘right to have access to’ operates via forms of reconnection between players, spaces and food, which sometimes occurs by means of more informal initiatives. Clouette compares two initiatives, one formal (Poiscaille, a sea *AMAP* or association supporting small-scale fishers) and the other informal (the *godaille*). In the first case, the producers are disconnected from their consumers because the boxes are sent to a Parisian clientele far from where the produce was caught. In the second case, the informal system of selling some of the catch to the fishers’ friends and neighbours improves access to high-quality fish for local populations and reconnects them both spatially and relationally with local fishing in a logic of proximity.

The relativity and contradictions inherent in food justice initiatives emphasise the extent to which the articles offer a critical perspective of ‘restorative’ initiatives for agrifood justice or solidarity. These initiatives are never satisfactory because they are neither multidimensional nor all-encompassing. They always act upon a single dimension of food systems: for example, land or consumption. Gallardo Gomez and Darrot reach the conclusion that the two initiatives they study do not manage to improve either participation or social inclusion because they are not systemic. If they are not food justice initiatives, this is perhaps also because agrifood justice represents neither their reference framework nor their objective. Instead, they are based on objectives to improve food accessibility or democracy. In this sense, adopting a systemic approach in food justice initiatives remains a challenge for both their design and their implementation.

Implicitly, therefore, the articles examine the operationalisation of food justice, what some call praxis, although the two concepts do not overlap. In fact, the praxis of food justice refers to the question of ‘how to do food justice’, characterised by a combination of theory, militancy and action. It implies that, if they are to be effective, it is crucial for actions to have a political dimension, in other words, attempt to influence the geometry of power and fundamentally transform the inequitable power relations within agrifood systems (Slocum et al., 2016). However, the case studies in these articles describe practices that are rarely or never underpinned by a clear theoretical framework from stakeholders. This is perhaps an additional point that explains why the initiatives struggle to achieve the objective of justice. The shift from practice to praxis is not yet operational in the initiatives studied.

Being in or out of the system to achieve agrifood justice is the final question we would like to address to conclude this reflection on agrifood justice as an inclusive process. Most of the food initiatives analysed in the articles are presented as alternatives, but are they within or outside of food systems? Do they necessarily need to be outside the system to be justice initiatives? Are they destined to remain confined to ‘micro-changes’ on the margins of policies, or are they part of a more global transformation of agrifood systems (Soulard & Duvernoy, 2017)? The answer is ambivalent. Proust explains that urban agriculture is a tool to circumvent dominant food systems, in particular because it allows indigenous varieties to be grown. In addition, informal micro-systems develop outside the dominant market. This is also true of Clouette's *godaille*. But can this be generalised? The discussion about the governance of agrifood justice and its institutionalisation deserves to be examined in depth, in a western context in which its classic implementation tools (food alternatives, urban agriculture, etc.) become models for public action in food or even social policies (e.g. the ‘Quartiers Fertiles’ operation that deals with the instrumentation of urban agriculture in France’s Urban Policy, cf. Tharreau, 2022).

## Conclusion: what would a just food transition involve?

In a context of global change, food and agriculture have become spearheads to implement transition. Food transition refers to the desired shift to a new, less energy-intensive and less polluting diet, which would make it possible to achieve self-sufficiency in order to reduce dependence on external supplies and markets. However, it is crucial to link these environmental issues about healthy and climate-smart food systems with the challenges of social sustainability and equity (Kaljonen et al., 2020). To what extent do the agrifood initiatives presented in this issue construct ‘transition pathways’ for food systems (Cohen & Ilieva, 2015)? The articles in this issue introduce some elements of discussion on the subject. They examine the way in which this concept is operationalised, in other words, what a just food transition would involve.

The studies make significant progress in the operationalisation of agrifood justice. First, they shed light on the nodes where injustices play out, such as the relationship with land, labour or exchange. These nodes are embodied in specific spaces or moments that are the expression of or even the medium for both relationships of domination and opportunities to ‘correct’ injustices: agricultural production spaces (Guillemin, Proust, Sosa et al.), processing and trading spaces (Guillemin, Clouette), and distribution and solidarity spaces (Gallardo Gomez & Darrot). These spaces are seen as spaces either of solidarity or domination, or as social spaces crossed by fault lines. Second, the articles provide an analysis of the processes of creating injustices but also actions or reconnections that make it possible to shift towards more justice, whether through the reappropriation of the act of production (Guillemin, Proust) or by the empowerment of traditionally minoritised groups in agrifood systems (Proust, Gallardo Gomez & Darrot).

Nevertheless, a systemic and global vision of all the components of food systems—essential to imagine a just food transition—has not yet been achieved and remains an ongoing field of research into how to operationalise agrifood justice.
